# Is visual activation associated with changes in cerebral high-energy phosphate levels?

**DOI:** 10.1007/s00429-018-1656-7

**Published:** 2018-03-23

**Authors:** Bart L. van de Bank, Marnix C. Maas, Lauren J. Bains, Arend Heerschap, Tom W. J. Scheenen

**Affiliations:** 1 Department of Radiology and Nuclear Medicine (766), Radboud University Medical Center, Geert Grooteplein-zuid 10, P.O. Box 9101, 6500 HB Nijmegen, The Netherlands; 2 Donders Institute for Brain, Cognition and Behaviour, Donders Centre for Cognitive Neuroimaging, Radboud University Nijmegen, Nijmegen, The Netherlands; 3 Erwin L. Hahn Institute, University Hospital Duisburg-Essen, Essen, Germany

**Keywords:** Functional magnetic resonance spectroscopy, Energy metabolism, In vivo ^31^P MRS, Human brain, Phosphocreatine (PCr)

## Abstract

Phosphorus magnetic resonance spectroscopy (^31^P MRS) has been employed before to assess phosphocreatine (PCr) and other high-energy phosphates in the visual cortex during visual stimulation with inconsistent results. We performed functional ^31^P MRS imaging in the visual cortex and control regions during a visual stimulation paradigm at an unprecedented sensitivity, exploiting a dedicated RF coil design at a 7 T MR system. Visual stimulation in a 3 min 24 s on–off paradigm in eight young healthy adults generated a clear BOLD effect with traditional ^1^H functional MRI in the visual cortex (average *z* score 9.9 ± 0.2). However, no significant event-related changes in any of the ^31^P metabolite concentrations, linewidths (7.9 ± 1.8 vs 7.8 ± 1.9 Hz) or tissue pH (7.07 ± 0.13 vs 7.06 ± 0.07) were detectable. Overall, our study of ^31^P MRSI in 15 cm^3^ voxels had a detection threshold for changes in PCr, Pi and γ-ATP between stimulation and rest of 5, 17 and 10%, respectively. In individual subjects, the mean coefficients of variance for PCr and Pi levels of control voxels were 6 ± 3 and 19 ± 8% (three time point average of 3 min 24 s). Altogether this indicates that energy supply for neuronal activation at this temporal resolution does not drain global PCr resources.

## Introduction

Phosphorus magnetic resonance spectroscopy (^31^P MRS) offers a non-invasive window on high-energy phosphates in the brain (Zhu et al. 2009). Substrates such as adenosine triphosphate (ATP) and phosphocreatine (PCr), as well as tissue pH can be monitored through the ^31^P MR spectra to provide a detailed view on the creatine kinase (CK) reaction: ${\text{PCr}}+{\text{ADP}}+{{\text{H}}^+}~\mathop \Leftrightarrow \limits^{{{\text{CK}}}} ~{\text{Cr}}+{\text{ATP}}$. Other ^31^P MRS visible phosphates that have a role in energy metabolism are inorganic phosphate (Pi), involved in ATPase reactions, and nicotinamide adenine nucleotides (NAD(H)), a substrate in redox reactions.

Contraction of muscles results in decreased PCr and increased Pi signals in their ^31^P MR spectra, reflecting the energy buffering role of PCr in muscles. Following these observations it was also attempted to measure such changes in the occipital area of the healthy human brain using visual stimulation (Sappey-Marinier et al. 1992; Kato et al. 1996; Murashita et al. 1999, 2000; Rango et al. 1997). However, these studies produced variable and non-reproducible results. Several report a (relative) decrease of PCr (Rango et al. 1997; Sappey-Marinier et al. 1992; Kato et al. 1996), while others do not observe this, in particular at the higher fields of 3 T and 4 T (Murashita et al. 2000; Barreto et al. 2014; Chen et al. 1997; Vidyasagar and Kauppinen 2008; Rango et al. 2006). Also (relative) increases in Pi and decreases in (NAD^+^ + NADH) signals upon visual stimulation are reported (Mochel et al. 2012; Barreto et al. 2014).

From a biological point of view, tissue-scale changes in the steady state levels of high-energy phosphates such as PCr and Pi are not expected to be very significant in the healthy brain as oxidative metabolism to provide ATP is dominating in this organ (Chen et al. 1997; Andres et al. 2008; Kemp 2000; Zhu et al. 2012). In contrast, simulation studies to understand the metabolic mechanisms underlying the BOLD phenomenon predict decreases in PCr content upon brain stimulation (Aubert and Costalat 2002).

The discrepancies in the observed changes in steady levels of high-energy phosphates of the brain upon visual stimulation call for further experiments with improved localization methods and higher MRS sensitivity using a stronger magnetic field and dedicated coil setups than used in previous studies. Recently, we developed a coil setup to perform ^31^P MRSI at 7 T with high spatial and temporal resolution within the human visual cortex (van de Bank et al. 2015). This setup consists of a ^31^P birdcage to excite the ^31^P spin system with uniform flip angles across the whole brain, combined with a 7-channel ^31^P array coil to maximize SNR in the occipital lobe. Sensitivity is further increased using whitened singular value decomposition for signal addition of individual receive channels (Rodgers and Robson 2016). Moreover, this setup is positioned within an eight-channel ^1^H head coil, enabling multi-transmit capabilities such as low-power saturation of the water signal for ^1^H–^31^P nuclear overhauser enhancement (NOE) of the ^31^P signals (Lagemaat et al. 2016). This enables us to dynamically measure ^31^P signals in 15 cm^3^ voxels with high temporal resolution within an activated region in the visual cortex.

The aim of this study was to use this optimized setup in dynamic ^31^P MRSI of the healthy human brain applying visual stimulation at durations which provide sufficient time for signal averaging but avoid habituation to investigate if changes occur in signals of ^31^P metabolites from voxels located in the BOLD-activated area.

## Materials and methods

### Subjects

A total of eight healthy volunteers (5 male and 3 female, mean age 33.0 ± 5.7 and 28.0 ± 3.5 years, respectively) with no neurological abnormalities participated in this study. Subjects were positioned head-first into the coil setup. When inside the coil, fixation cushions were used to minimize head motion. A mirror was placed in front of the eyes to enable the subject to view the complete projection.

## Compliance with ethical standards

### Ethical approval

All procedures performed in this study involving human participants were in accordance with the ethical standards of the human ethics committee of the Medical Faculty of the University of Duisburg-Essen and with the 1964 Helsinki declaration and its later amendments.

### Informed consent

Informed consent was obtained from all individual participants included in the study.

## Data acquisition

### Hardware

All experiments were performed on a 7 T whole body MR system (Magnetom 7T, Siemens Healthcare, Erlangen, Germany) with a home-built coil setup, consisting of an 8-channel ^1^H transceiver array around the whole head, an actively detunable ^31^P volume resonator and a dedicated ^31^P 7-channel receive-only array at the occipital region of the brain (van de Bank et al. 2015).

### MR protocol

**Fig. 1:**
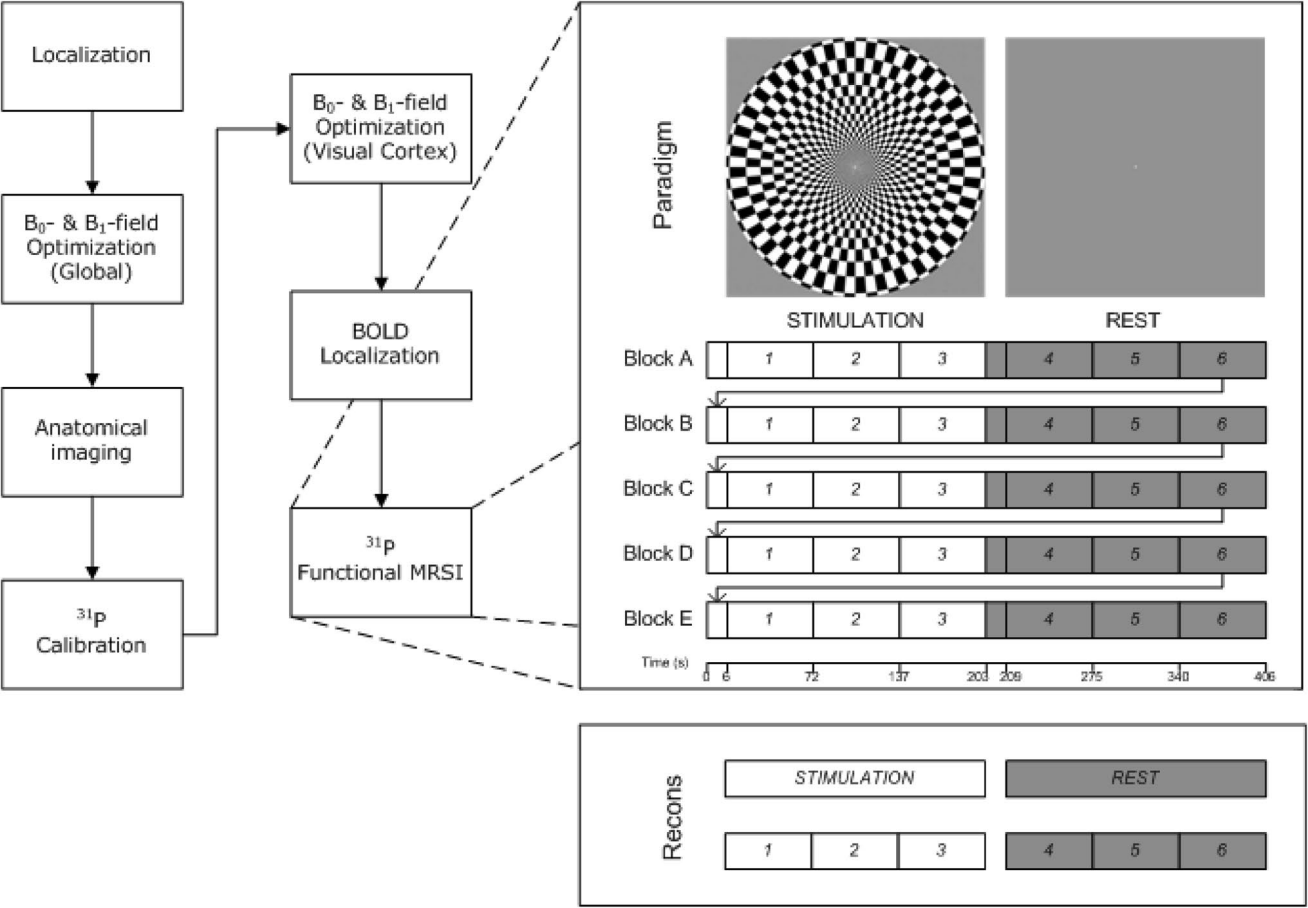
Schematic measurement protocol. After calibration and pre-scans, the ^31^P functional MRSI consisted of three examinations (68 s each) during visual stimulus, followed by three acquisitions during rest. This ^31^P MRSI sequence was repeated up to five times

The complete protocol for this study is presented in Fig. 1 and could be performed within 1.5 h. Within this protocol, an anatomical T1-weighted 3D magnetization prepared rapid gradient echo sequence (MPRAGE) was acquired with B_0_- and B_1_-shims optimized for the whole brain. The parameters for this anatomical image were: TR/TI/TE = 2500/1100/1.35 ms, flip angle 6°, FOV 176 × 224 × 192 mm^3^, resolution 1 mm^3^, acquisition time (Tacq) 3 min 58 s.

With a BOLD localizer and an on–off visual stimulation paradigm, the region of activation within the visual cortex was determined. This scan was used to select voxels from the 3D ^31^P MRSI inside and outside the activated region. Parameters of the gradient echo EPI BOLD localizer: TR/TE 3000/54 ms, flip angle 90°, FOV 263 × 263, slice thickness 1.8 mm, number of slices 33, voxel size 2.1 × 2.1 × 1.8 mm^3^, measurements 120, on–off paradigm pairs 10, bandwidth 2442 Hz/pixel, EPI factor 128, Tacq 6 min 9 s.

The T1-weighted images were used for positioning the shim volume centered on the visual cortex and for co-localization of the ^31^P-MRSI grid with the same region. After adjusting the B_0_-shims for the occipital lobe, the power of the 90° flip angle for ^31^P was calibrated with six slice-selective ^31^P excitations in steps with varying pulse voltage. With B_1_ shimming the proton transmit field was homogenized over the occipital lobe for localized homogeneous NOE enhancement, locally increasing ^31^P sensitivity (Lagemaat et al. 2016).

With all experimental parameters optimized, the pulse-acquire ^31^P-fMRSI examination during the visual stimulation paradigm was performed with the following parameters: TR 400 ms, matrix size 8 × 8 × 8, FOV 120 × 120 × 120 mm^3^, acquisition-weighted elliptical *k*-space sampling with three averages of *k*-space center, hamming filter apodization of all *k*-space dimensions resulting in a spherical voxel size of 15 cm^3^, six preparation scans, flip angle 25° (Ernst angle for PCr at this TR), pulse duration 0.3 ms (β-ATP not fully excited by this hard pulse, not used for further analysis), Tacq for one full ^31^P-MRSI data set: 1 min 8 s. The subject was asked to focus at the center of the projection screen during the examination, where repeatedly either a flickering checkerboard or nothing was projected during each block of 3:24 min (Fig. 1).

NOE enhancement was generated by saturating the water signals (γB_1_ = 30 Hz) using a wideband alternating phase low-power technique (derived from WALTZ-4 technique, originally intended for decoupling) (Shaka et al. 1983) during the full TR, except during the 146 ms of signal acquisition.

### Paradigm

The visual stimulation consisted of a black–white radial checkerboard (Fig. 1) pattern flickering at 8 Hz, programmed using presentation (Neurobehavioural Systems, Berkeley, USA). It was projected onto a screen inside the magnet bore behind the coil setup. Subjects viewed the visual display via a mirror mounted inside the head coil right in front of their eyes. For the BOLD localizer, the paradigm was repeatedly turned on and off in 18 s intervals, starting with a rest measurement. The paradigm for the ^31^P-MRSI started with displaying a checkerboard for 3 min and 24 s (duration of three consecutive ^31^P MRSI data acquisitions), followed by a rest period with the same duration (Fig. 1). The duration of the paradigm was minimized to avoid habituation. This stimulation protocol was repeated up to five times (approximately 34 min, or shorter with less repeats if requested by the volunteers).

### Data analysis

**Fig. 2:**
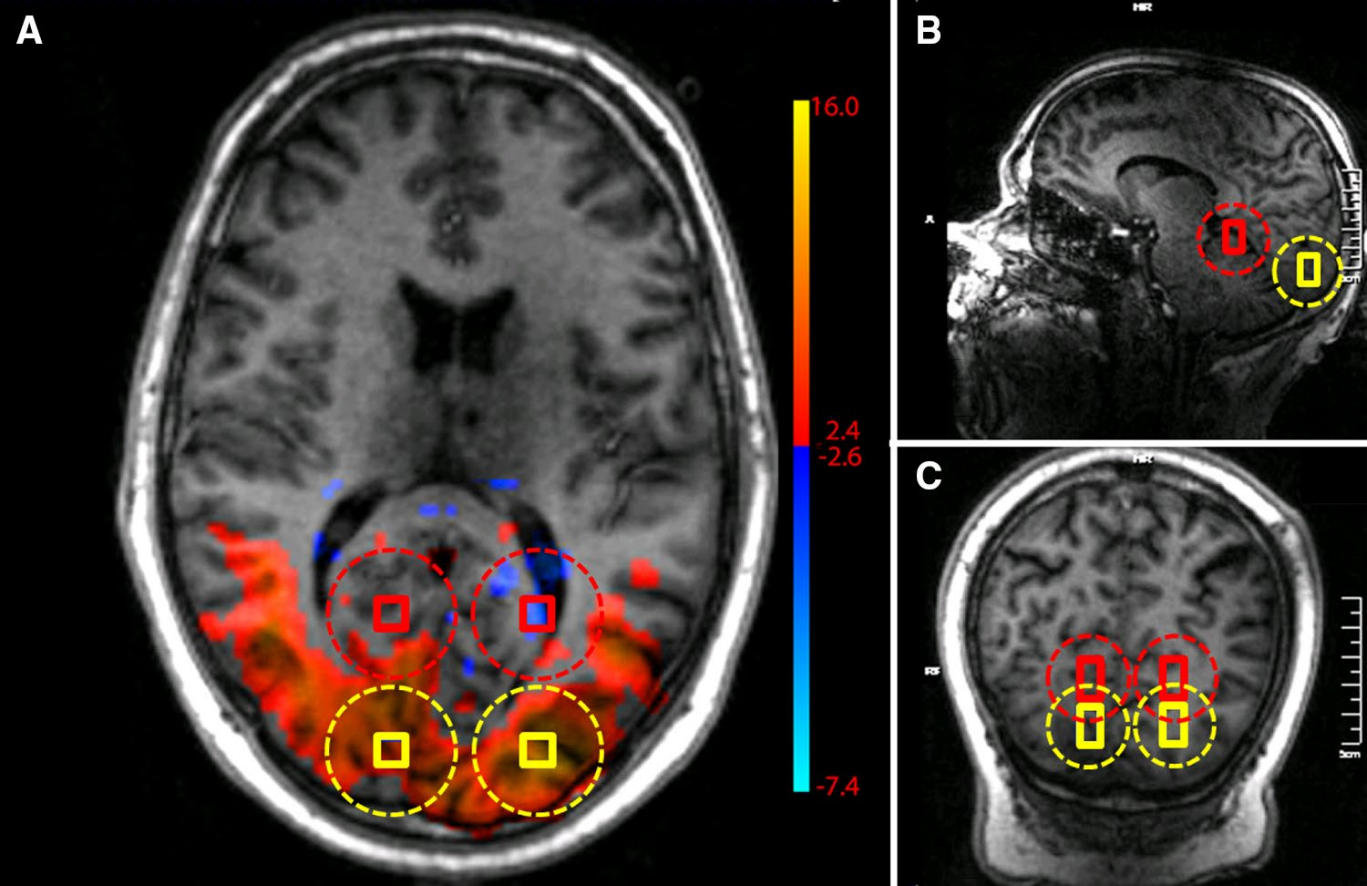
**a** Transverse, **b** sagittal and **c** coronal slices of the anatomical image of one of the volunteers overlaid with *z* score maps from the BOLD localizer. Also shown is the co-localization of the selected voxels from 3D ^31^P-MRSI with the activated region (yellow) and the control voxels outside the activated region (red). The dotted circle shows the true voxel size. The average *z* score for the spectroscopy voxels within the activated region of visual cortex was 9.9 ± 0.2 as determined by the independent component analysis

For data analysis, one 15 cm^3^ voxel in the BOLD-activation region in the visual cortex in the right hemisphere and one in the left hemisphere were selected to measure phosphorus metabolite levels, likewise two voxels outside the visual cortex were selected as controls (Fig. 2). Voxels were chosen to be spatially independent from each other, even after taking into account Hamming filtering of k-space of the 3D ^31^P-MRSI data, which increases voxel size (Scheenen et al. 2008).

Multiple ^31^P-MRSI datasets were acquired during the stimulation paradigm. The selected voxels were extracted from the datasets using Matlab 2014b (Mathworks, Natick, MA, USA) and ported into the jMRUI software package (version 5.0) (Stefan et al. 2009; Naressi et al. 2001). In this program, each spectrum of the selected voxels was phase and frequency aligned. For quantification, the AMARES fitting algorithm was used (Vanhamme et al. 1997). The fitted integrals and linewidths of the ATP, PCr, Pi, PME, PDE and NAD(H) signals were obtained and further analyzed using Matlab. The pH was determined from the chemical shift difference between the PCr and Pi resonance peaks (Petroff et al. 1985). To examine possible changes in high-energy phosphate levels during stimulation, data were not only averaged per stimulation block, but also per acquisition point within a block (three per block, six per paradigm repetition), i.e., all of the first acquisitions during stimulation were averaged, all of the second acquisitions, etc. (Fig. 1). To combine the data from all subjects, fitted signal integrals were normalized by their mean value over time and weighted according to the number of blocks obtained in the subject.

### Statistics

Descriptive parameters were calculated and presented as mean ± standard deviation (SD). A paired two-tailed Student’s *t* test was used to compare the integrals of the metabolites during stimulation and during rest. A *p* value of *p* < 0.05 was considered to be statistically significant.

To investigate stability and quality of the spectra over time from each subject individually, we determined the coefficient of variation (CoV)

$${\text{Co}}{{\text{V}}_{s,m}}=\frac{{{\sigma _x}}}{{{{\bar {\mu }}_x}}} \times 100\% ,$$

where ${\sigma _x}$ represents the SD of the metabolite per voxel per subject, ${\bar {\mu }_x}$ the mean signal of the metabolite per voxel per subject, and ${\text{Co}}{{\text{V}}_{s,m}}$ the CoV per metabolite (*m*) per subject (*s*), which is reported as a mean percentage per metabolite over the different subjects. By definition, 95% of the measured signal integrals in one voxel of a subject resides within plus or minus two times the CoV around its mean.

In a post hoc power analysis for paired differences with a two-sided significance level of 5% and a power of 80% of all blocks of all volunteers, the detection limits of changes in normalized PCr, Pi and γ-ATP signal integral were calculated, which is a measure for the smallest reliably detectable change in signal integral to be estimated from all data combined.

## Results

**Fig. 3:**
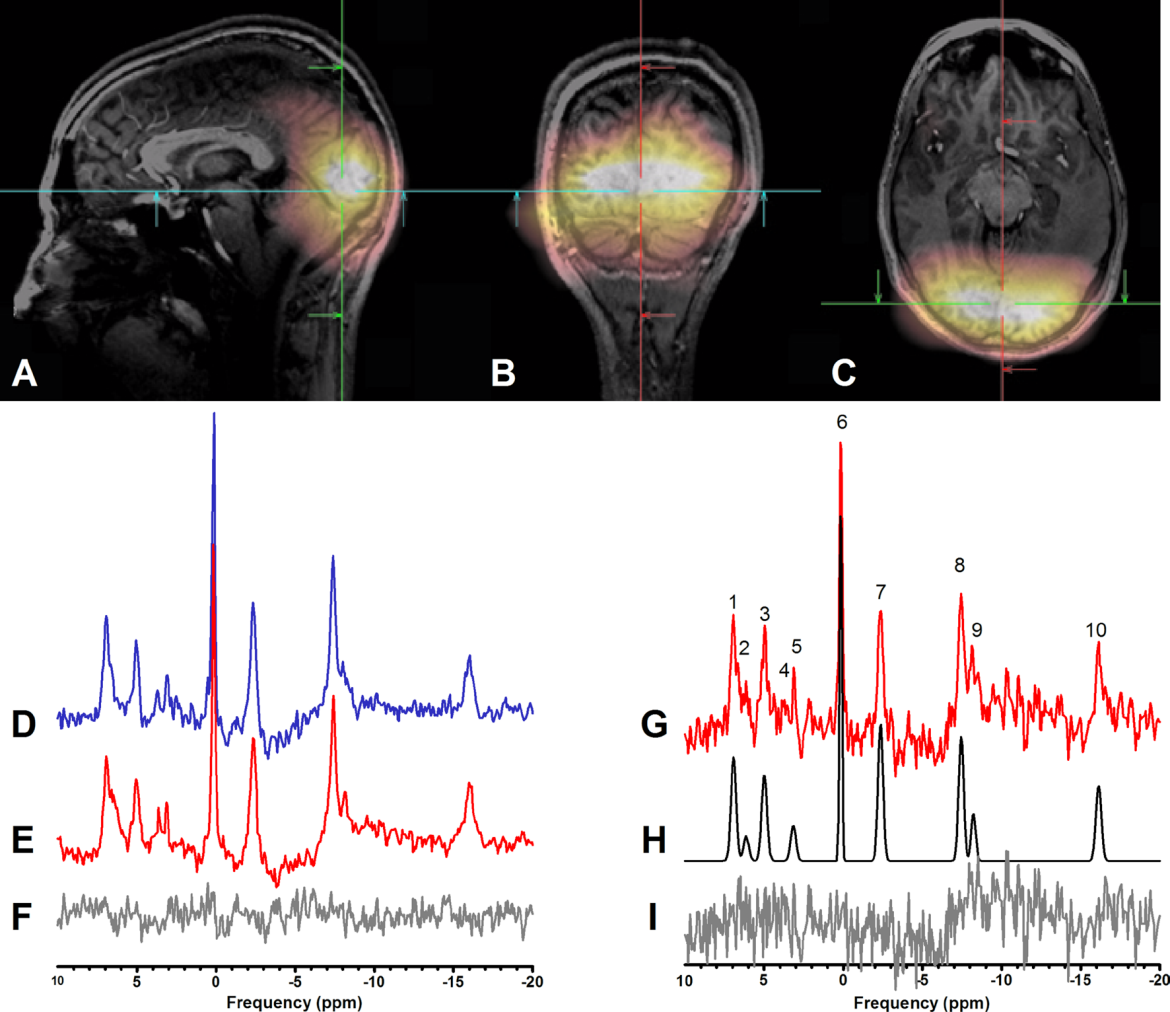
Sensitivity profile and spectral quality of the measurement setup. The ^31^P coil array sensitivity profile is restricted to the occipital lobe of the human brain, as shown by PCr images overlaid on a sagittal (**a**), coronal (**b**), and transversal (**c**) reconstruction of the 3D MPRAGE images. ^31^P spectral quality from voxels in the visual cortex of a single subject is reflected in **d** averaged spectra (one voxel, 5 blocks of 3 time points) during stimulation, **e** averaged spectra during rest and **f** difference between stimulation and rest. For comparison in **g** the spectrum of one voxel at one time point (68 s acquisition) from the same volunteer is presented. The fit as done by jMRui is shown in **h** with **i** the residuals. Note that spectra were subtracted without additional line broadening of the resting state spectra to compensate for BOLD effects (Mangia et al. 2007). The metabolites identified in these spectra are (1) phosphoethanolamine (PE), (2) phosphocholine (PC), (3) Pi, (4) glycerophosphoethanolamine (GPE), (5) glycerophosphocholine (GPC), (6) PCr, (7) γ-ATP, (8) α-ATP, (9) NADH + NAD^+^ and (10) β-ATP

The optimized coil setup, separating transmit and receive of ^31^P signals, enabled us to use short-hard excitation pulses in a short repetition time to acquire 3D ^31^P MRSI spectra in the human visual cortex in 68 s. The quality of individual spectra was sufficient for analysis of the resonances of interest. In these spectra (Fig. 3), the following metabolites were quantified with a Cramer–Raó lower bound (CRLB) < 30%: PCr, γ-ATP, α-ATP, β-ATP and Pi (Table 1). The signals of other metabolites were not further evaluated, either because their CRLB was < 30% for less than 10% of the spectra [e.g., NAD(H) and phosphodiesters] or because no changes by visual stimulation were expected. In five subjects, the complete MRSI examination (10 blocks, 30 scans) could be performed, in the three other subjects data acquisition was stopped, respectively, after 24 scans (8 blocks), after 12 scans (4 blocks) and after 6 scans (2 blocks). Linewidths of PCr were 7.9 ± 1.8 and 6.8 ± 0.8 Hz for the visual cortex and the control voxel, respectively.

**Table 1:** Measurement accuracy and stability of high-energy ^31^P metabolites

			PCr	Pi	γ-ATP	α-ATP
Metabolite levels	Detected	Visual cortex	100	76	100	86
		Control	100	81	100	92
	Mean CRLB	Visual cortex	4	20	9	16
		Control	4	19	9	14
CoV	Short term	Visual cortex	11 ± 7	33 ± 8	23 ± 9	25 ± 8
		Control	11 ± 4	32 ± 8	25 ± 9	23 ± 8
	Long term	Visual cortex	6 ± 5	20 ± 8	12 ± 5	15 ± 6
		Control	6 ± 3	19 ± 8	12 ± 6	13 ± 7

384 spectra were used for analysis for both locations. The percentages of metabolites fitted with CRLB < 30% are shown for both locations, with the corresponding mean CRLB (calculated from values < 30%). The coefficient of variation (CoV) is calculated in two different ways, a short-term CoV [for the individually acquired spectra (68 s)] and long-term CoV [for the separate blocks, averaged over three time points per block (3 min 24 s)]. All values are given as percentages

**Fig. 4:**
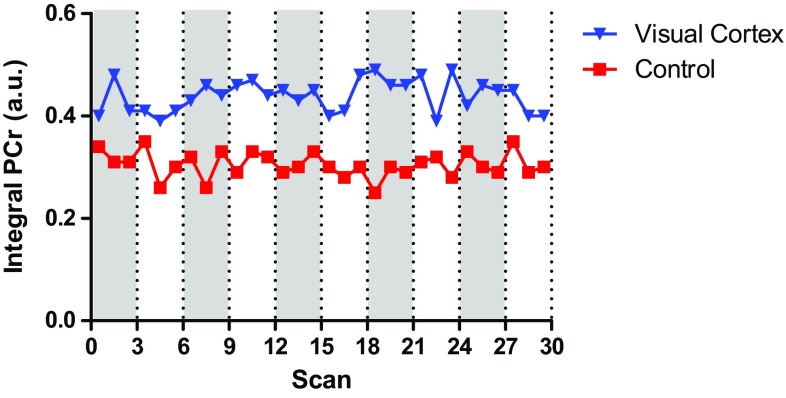
Integral of the fit to the PCr signal over time of a single subject for a voxel located at the visual cortex (CoV 7%) and for a control voxel (CoV 8%). The paradigm started with three stimulation measurements (gray), followed by three rest (white) measurements

The overall stability of the ^31^P MRSI data acquisitions was assessed by the CoV of the largest signal (PCr) over time for both the control and visual cortex voxels in each subject. The mean CoV for 68-s PCr signal integrals in control voxels of the eight volunteers was 11 ± 4% (Table 1). Systematic variations in individually acquired 68-s spectra need to be greater than twice this CoV of 11% for PCr and 33% for Pi to be detected in one volunteer. Variations in PCr and Pi greater than 12 or 40% (twice the long-term CoV for both metabolites) would be detectable in an individual volunteer when assessed between stimulation and rest periods (3-time point averages over 3 min, Table 1). Individual integral values of PCr for a single subject (CoV 7%) showed that some fluctuations seemed to coincide with the applied paradigm (Fig. 4), but they did not systematically extend outside twice the CoV.

**Fig. 5:**
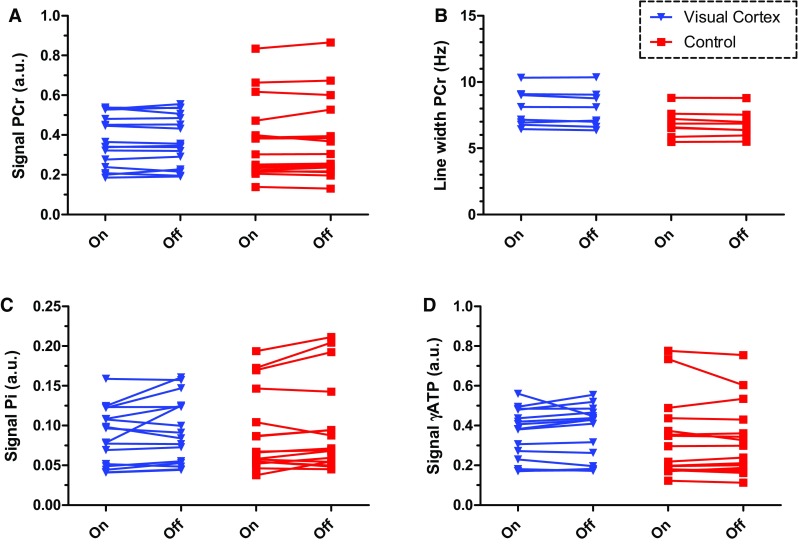
Paired analysis of concentrations of PCr, Pi and γ-ATP with and without visual stimulation from the selected voxels within the visual cortex (blue, triangle) and the control area (red, square) of all eight volunteers (two voxels in each area, 16 pairs). **a** PCr signal integral during rest (3-time point average within a block) and during stimulation (3-time point average within a block). **b** Linewidth of the PCr signal with and without visual stimulation. **c, d** Signal integrals of Pi and γ-ATP, respectively, with and without visual stimulation

We did not find significant differences in any of the integrals or linewidths of the metabolites of interest between stimulation and rest periods. In voxels in the visual cortex, the three time point averages of the PCr signal integral did not change during visual stimulation compared to rest (*p* values of paired analysis 0.98, Fig. 5). Control voxels were not significantly different either (*p* = 0.37 for PCr signal integral). The PCr linewidths did not change because of activation (7.9 ± 1.4 vs 8.0 ± 1.4 Hz, *p* value of paired analysis 0.16) and remained unchanged in the control voxels (6.8 ± 1.0 vs 6.9 ± 1.0 Hz, *p* value of paired analysis 0.13). Subtracting all rest spectra from the stimulation spectra revealed no changes in PCr either (Fig. 3f). No significant changes were detected for Pi or for γ-ATP signal integrals between stimulation and rest periods (visual cortex *p* = 0.10 and *p* = 0.45, respectively, and *p* = 0.08 and *p* = 0.44 for the control voxels, Fig. 5c, d). Moreover, the variations in signal integrals in time between voxels located within the visual cortex compared to control voxels were comparable (Table 1). The pH was also stable during the stimulation protocol, its mean value during stimulation in the visual cortex was determined at 7.07 ± 0.13 (7.04 ± 0.09 in control) and during rest at 7.06 ± 0.07 (7.06 ± 0.10 in control). Even in the subject with the smallest spread in pH values of 0.04 (SD) at the control locations, in which no changes with respect to the paradigm were expected, the SD at the two selected voxels in the BOLD-activated region was similarly small 0.03, indicating the absence of a pH effect larger than 0.06 (2 × SD) in the most repeatable measurement.

The post hoc power analysis of 64 paired paradigm blocks of all eight volunteers resulted in a detection limit for an event-related PCr, Pi or γ-ATP signal integral change of 5, 17 and 10%, respectively. If the PCr signal integral was systematically modulated either up or down by 5% (or more) by the visual stimulation paradigm, we would have detected it.

**Fig. 6:**
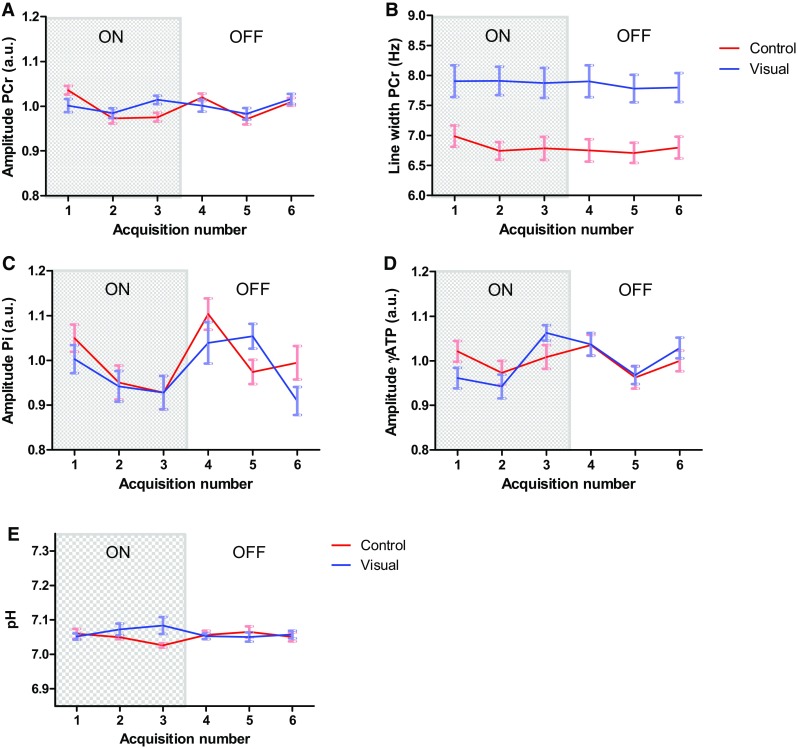
Evolution of normalized signals of all eight volunteers during stimulation (first three acquisitions) and during rest periods (last three acquisitions). **a** Changes in group average of the signal amplitude of PCr during the paradigm and **b** changes in linewidth of the PCr were small and not event-related. **c** The changes in group average of the normalized signal amplitude of Pi and **d** for γ-ATP. **e** Block-weighted average pH during the stimulation and rest periods. The mean pH value during the stimulation was 7.07 ± 0.13 (SD over all blocks), and during rest 7.06 ± 0.07 in the visual cortex. All error bars are standard errors

Our data acquisition strategy allowed another possibility for data analysis, evaluating temporal changes within stimulation and rest blocks of several interesting ^31^P metabolites. Normalized individual acquisitions within the paradigm were averaged across different repetitions of the paradigm (thus, first acquisition during stimulation in first block with the first acquisition of the second block, etc.) (Fig. 1) and across all volunteers. This analysis showed subtle variations in averaged signal of PCr, but none were statistically significant (Fig. 6a). Again, the linewidth of PCr did not change over time during and between the stimulation and rest periods (Fig. 6b).

The same analysis was performed for Pi and for γ-ATP. Both time courses were also normalized by the mean signal of each metabolite for each subject, before taking the weighted average. For Pi, just as for γ-ATP, no event-related differences between the voxels in the visual cortex vs controls were found (Fig. 6c, d). The plot of the time course for pH (block-weighted average of all volunteers) did not show any differences beyond the standard deviations of the individual acquisition numbers within the on–off events, and no event-related change beyond twice the standard error (Fig. 6e).

## Discussion

In this study, we monitored the levels of high-energy phosphates in the human visual cortex with dynamic ^31^P MRSI at 7 T with a temporal resolution of 68 s during an intensive visual stimulation (VS) paradigm. Using an optimized RF coil setup together with NOE enhancement, the sensitivity of these measurements was maximized as reflected in the low CoVs of the assessments of repeated signal integrals. The MRSI data revealed that neither the levels of the high-energy phosphates nor the pH significantly changed in the activated visual cortex during visual stimulation.

Functional imaging of the brain aims to measure events related to triggered action potentials. Initiating these events and re-establishing equilibrium, costs energy that has to be produced by, or delivered to, the cells in action (Harris et al. 2012). So far, capturing the BOLD effect has played a central role in measuring brain function, which is an indirect way to measure energy expenditure of the human brain. A more direct approach is the dynamic measurement of tissue concentrations of high-energy phosphates as done in this study.

It is important to measure these concentrations accurately and with high reproducibility, which is challenged by several instrumental factors. One of these is the use of a dedicated RF coil, which directly influences measurement accuracy by its spatial coverage and its sensitivity. Although localization by surface coils achieves an excellent SNR and could provide a high temporal resolution, partial volume effects and additional spatial weight of tissue signals because of the reception profile will be present, and further localization is needed to target the activated region within the visual cortex. A coil’s sensitivity is mainly determined by its RF profile, especially when the same coil is used for signal transmission and reception. The RF coil setup developed for this ^31^P study consisted of separate transmit and receive parts for which relatively small coil elements were used to increase receive sensitivity in the occipital lobe of the brain (van de Bank et al. 2015). When combined with localization by phase encoding (3D MRSI), it allows us to obtain specific spectral information from the visual cortex. Moreover, signal intensities of high-energy phosphates were further enhanced using NOE, without reducing repeatability (Lagemaat et al. 2016). Together this enabled dynamic monitoring of high-energy phosphate levels during visual stimulation at both high spatial resolution (15 cm^3^) and high temporal resolution (68 s). Our approach in the short TR regime might make the measurements vulnerable to dynamic changes in T1 relaxation of the metabolites of interest, if these changes occur when metabolites cycle faster through the enzymatic reactions of creatine kinase and ATPase (Kim et al. 2017).

A potential problem in the analysis of MRS data obtained from BOLD-activated areas is a decrease in linewidth due to a change in local susceptibility by a decrease of deoxyhemoglobin in blood supplying this area. In visual stimulation experiments analyzed by ^1^H MRS performed at 7 T, this was taken into account by a line broadening of 0.48 Hz of the spectra at stimulation before subtraction from the rest spectra (Mangia et al. 2007). If we would apply this to the ^31^P MR spectra the additional broadening would be in the order of 0.19 Hz $[0.48/({\gamma _{^{1}{\text{H}}}}/{\gamma _{^{31}P}})]$ and therefore negligible (maximum influence is only 4%). Furthermore, no significant differences in linewidths between both states were detected (Fig. 5b), demonstrating that indeed the BOLD effect has limited influence on the ^31^P signals.

An implicit measure obtained from this study is the detection threshold for paradigm-induced variations in high-energy phosphate metabolite levels. Variations in these levels are only detected when they are greater than the relative dispersion of the data, as reflected by the CoV of the individual volunteers. In this work, none of the parameters of interest exhibited detectable event-related changes. The overall detection threshold for event-related changes in the PCr signal intensity in eight volunteers in the visual cortex was 5%: a change larger or equal to 5% would have been detected in this work.

## Relation to previous studies

In several studies, ^31^P MRS has been employed to evaluate changes in brain metabolite levels during VS in healthy volunteers. Most of these present a (relative) decrease of PCr levels, although the results are not very consistent (Sappey-Marinier et al. 1992; Kato et al. 1996; Murashita et al. 1999; Rango et al. 1997; Barreto et al. 2014). The earliest study, performed at 2.0 T, reported a decrease of about 40% in the ratio PCr/Pi in a 32 mL voxel centered at the visual cortex over a 12.8 min period of VS (Sappey-Marinier et al. 1992). Another study at 1.5T showed that the ratio of PCr/(∑^31^P-metabolites) decreased by about 11% over a 12 min VS period (Kato et al. 1996). The same group later reported a decrease of about 17% in concentration of PCr during 6 min of VS, but only for men over 40 years of age; in younger volunteers PCr did not drop significantly (Murashita et al. 1999). Another 1.5T study reported a PCr decrease of about 18% recorded in young adults immediately after a VS of only 3.5 s (Rango et al. 1997), but the same group reported later that during a VS of 7 min PCr did not decrease, but pH increased and in the recovery period the sum of PCr and βATP increased (Rango et al. 2006). In a more recent study at 4T, a decrease of PCr and increased pH was observed in young healthy adults after 12 min of VS (Yuksel et al. 2015). In apparent agreement with these results, simulation studies to understand the metabolic processes underlying the BOLD phenomena, suggested that a substantial decrease in the brain levels of PCr upon stimulation could be expected (Aubert and Costalat 2002).

However, in a different study on healthy volunteers at 4T, no decrease in PCr by VS was seen (Chen et al. 1997). Also, no change in PCr and tissue pH was observed at 3 T by a VS of about 7 min, even under mild hypoxic conditions (Vidyasagar and Kauppinen 2008). A ^31^P MRS study with ^1^H decoupling and NOE of the healthy brain of young adults performed at 3T with VS for 1.5 or 5 min also did not record a change in PCr and pH (Barreto et al. 2014). Our results are in complete agreement with the latter observations, no effect on PCr and pH by VS was observed, even by monitoring the visual cortex with the currently most sensitive ^31^P MRSI conditions, i.e., at 7T with a phased-array ^31^P receive coil and NOE. This indicates that at the temporal resolution of our experiment tissue PCr levels are not an important energy resource for the brain of young healthy adults during visual stimulation. Our results are more in agreement with a highly oxidative energy supply during brain activation, with perhaps a role for PCr in facilitated diffusion of high-energy phosphates from the intracellular production to consumption site (Chen et al. 1997; Zhu et al. 2012; Lee et al. 2014; Mangia et al. 2007).

Next to reports focusing on changes in PCr and pH by VS, some studies reported on changes in other ^31^P resonances. For instance, a study at 3T inferred an increased Pi from increased Pi/PCr (11%) and Pi/ATP (13%) ratios during an 8 min VS period in a group of healthy middle aged volunteers (Mochel et al. 2012). Also at 3 T, an increased relative Pi of 15 and 3% and a decreased NAD(H) level of 5 and 2% was reported in young healthy adults for stimulation blocks of 1.5 and 5 min, respectively (Barreto et al. 2014). The study did not show significant event-related changes in Pi/PCr ratio. The time-dependent decrease was attributed to a habituation effect. These reported changes (relative to ATP) are below the detection threshold for Pi of the current study and can therefore not be confirmed or dismissed here. The same holds for the NAD(H) results, as in the majority of our localized MR spectra the fit of the NAD(H) peaks did not meet our CRLB criteria. The reported accuracies of these earlier studies seem remarkably high for coil-localized excitation without a control area at 3 T.

The difference between our results and those reported by others and also among the ^31^P MRS studies of VS may be due to variations in the duration and type of visual stimulation, to the use of low field MR systems (1.5 T) with insufficient chemical shift dispersion and to partial volume effects because of the use of surface coils without further localization of the visual cortex or of the area showing a BOLD effect. What often lacks in the existing literature is the inclusion of a signal from a voxel or surface coil outside the visual cortex as a control signal in the same subject.

Another potential reason for variable results may be age. The majority of the studies discussed above involved young healthy adults, but a different effect on PCr between younger and older men has been observed (Murashita et al. 1999). A potential age effect in the response to VS needs further exploration. Several ^31^P MRS studies also reported that diseased brains responded differently to a VS paradigm than healthy brains, such as in bipolar disorders (Yuksel et al. 2015), Huntington (Mochel et al. 2012; Adanyeguh et al. 2015) and mitochondrial disorders (Kato et al. 1998).

Our activation paradigm focused on interrogating the role of high-energy phosphates in intensive brain activation. Habituation resulting from the prolonged activation was not expected with our stimulation protocol, where other studies have applied VS for more than 10 min (Mangia et al. 2007; Sappey-Marinier et al. 1992; Kato et al. 1996; Yuksel et al. 2015). To examine if quick temporal changes in concentrations occur at the initialization of a new state within the visual paradigm, a much higher temporal resolution is needed to confirm the 18% decrease in PCr level in a stimulation protocol of only 3.5 s (Rango et al. 1997). This cannot be achieved by standard 3D MRSI, but may be possible by solely relying on localization by surface coils, similar to other studies (Lee et al. 2014; Sappey-Marinier et al. 1992; Rango et al. 1997; Chen et al. 1997; Kato et al. 1996; Murashita et al. 1999) or by applying a triggered activation protocol as performed in ^31^P MRS studies of muscles (Kan et al. 2009).

In our experiments, one can observe minor fluctuations in PCr signal intensity during visual activation, but none of them were statistically significantly related to the stimulation paradigm. Even in the subject with the lowest CoV in the control voxels in which no event-related changes are expected (the ‘best’, most repeatable examination in our study), we did not see any significant differences in signal integrals related to the paradigm in the voxels of the visual cortex, which means that if these differences were present, they were below the detection threshold of 8% for this individual (twice the CoV for those voxels). All our observations refer to global PCr levels (sum of all PCr present in a 15 cm^3^ voxel), it may very well be that at the cellular level PCr still functions as a local temporal energy buffer (Andres et al. 2008). The absence of changes in high-energy phosphates and pH during VS does not imply that they are not involved in brain energy metabolism in this activity. Their role has been elegantly demonstrated in magnetization transfer experiments; VS-related increases in the forward fluxes of both the creatine kinase and ATPase reactions have been reported, indicating enhanced phosphate cycling and oxidative energy supply to match the needs of increased brain activity (Lee et al. 2014; Chen et al. 1997). In the study performed by Lee et al., the intrinsic higher SNR of an ultra strong magnetic field (7 T) was exploited to measure these fluxes.

## Conclusion

In this study, no change was observed for high-energy phosphates and pH in the visual cortex of young healthy adults during an intensive photic stimulation paradigm above the detection threshold, which was 5% for PCr, 17% for Pi and 10% for γ-ATP. Previous reports of changes in PCr or pH on a timescale of minutes during visual stimulation could not be reproduced or were below our detection limits (Pi and ATP) even with a dedicated technical setup at ultra-high magnetic field strength with maximized sensitivity. On this timescale of minutes, and although it is involved in rapid cellular energy distribution, PCr does not seem to serve as a global energy buffer.
